# Serpin Inhibition Mechanism: A Delicate Balance between Native Metastable State and Polymerization

**DOI:** 10.4061/2011/606797

**Published:** 2011-05-24

**Authors:** Mohammad Sazzad Khan, Poonam Singh, Asim Azhar, Asma Naseem, Qudsia Rashid, Mohammad Anaul Kabir, Mohamad Aman Jairajpuri

**Affiliations:** ^1^Department of Biosciences, Jamia Millia Islamia University, Jamia Nagar, New Delhi 110025, India; ^2^Department of Biotechnology, National Institute of Technology Calicut (NITC), NIT Campus P.O., Calicut, Kerala 673601, India

## Abstract

The serpins (**ser**ine **p**roteinase **in**hibitor**s**) are structurally similar but functionally diverse proteins that fold into a conserved structure and employ a unique suicide substrate-like inhibitory mechanism. Serpins play absolutely critical role in the control of proteases involved in the inflammatory, complement, coagulation and fibrinolytic pathways and are associated with many conformational diseases. Serpin's native state is a metastable state which transforms to a more stable state during its inhibitory mechanism. Serpin in the native form is in the stressed (S) conformation that undergoes a transition to a relaxed (R) conformation for the protease inhibition. During this transition the region called as reactive center loop which interacts with target proteases, inserts itself into the center of **β**-sheet A to form an extra strand. Serpin is delicately balanced to perform its function with many critical residues involved in maintaining metastability. However due to its typical mechanism of inhibition, naturally occurring serpin variants produces conformational instability that allows insertion of RCL of one molecule into the **β**-sheet A of another to form a loop-sheet linkage leading to its polymerization and aggregation. Thus understanding the molecular basis and amino acid involved in serpin polymerization mechanism is critical to devising strategies for its cure.

## 1. Introduction

Serpins (**ser**ine **p**roteinase **in**hibitor**s**) are the largest super family of protease inhibitors involved in many critical biological processes like blood coagulation, fibrinolysis, programmed cell death, development and inflammation [1–3]. Serpins are structurally heterogeneous and functionally diverse proteins found in the organisms ranging from viruses to vertebrates [4–6]. Over 400 different serpins have been identified in organisms including viruses, plants, insects, animals and prokaryotes [7]. Serpins like *α*-antitrypsin, *α*-antichymotrypsin, C1-inhibitors, antithrombin, and plasminogen activator inhibitor-1 (PAI), play absolutely critical role in the control of proteases involved in the inflammatory, complement, coagulation and fibrinolytic pathways, respectively, and are associated with diseases like emphysema/cirrhosis, angioedema, familial dementia, chronic obstructive bronchitis and thrombosis [8]. Not all serpins act as proteinase inhibitors some are inhibitors of other types of proteinases, while others are noninhibitors. For example, the viral serpin crmA inhibits interleukin-1 converting enzyme and squamous cell carcinoma, antigen-1 (SCCA-1) inhibits cysteinyl proteinases of the papain family. Noninhibitory serpins perform diverse functions, including roles as chaperones, for example, the 47-kD heat shock protein [HSP47] and hormone transportation like cortisol-binding globulin [7]. 

## 2. Serpin Structure and Common Mechanism of Action

**Figure 1:**
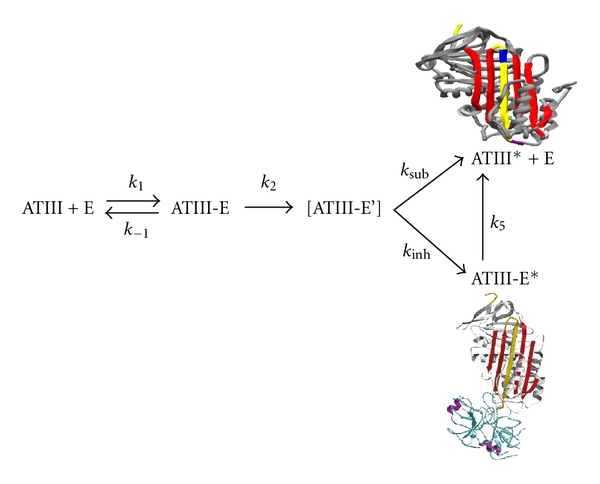
The scheme shown here (taking antithrombin as an example) represents the suicide substrate inhibition mechanism common to all inhibitory serpins. The scheme represents the interaction between the serpin (antithrombin, ATIII) and protease (E); ATIII-E is the noncovalent Michaelis complex; ATIII-E' is the proposed intermediate before partitioning; ATIII-E* is the stable protease-inhibitor complex; ATIII* is the cleaved ATIII. The outcome of the reaction is dependent on the partitioning between the inhibitory (*k *_inh_) and substrate pathways (*k *_sub_). The figures represent the cleaved and factor Xa bound ternary complexes of antithrombin [3].

There is a high rate of conservation in the structure among the members of serpin family in which the average size of protein is 350–400 amino acids and the molecular weight of 40–50 kDa [4]. The serpin fold is comprised of 3-*β* sheets (A, B, C) and 7–9 alpha helices. A solvent exposed stretch of amino acids termed as reactive centre loop (RCL) contains the protein recognition site which forms a flexible stretch of ~17 residues between *β* sheets A and C [8]. The mechanism of inhibition of serpin has been demonstrated biophysically and structurally as suicide substrate-like inhibitory mechanism (as shown in Figure 1) where after binding to protease it is partitioned between cleaved serpin and serpin-protease complex [9]. Initially serpin binds to protease through a noncovalent Michaelis-like complex by interactions with residues flanking the scissile bond (P1-P1′) [6]. Attack of the active site serine on the scissile bond leads to a covalent ester linkage between Ser-195 of the protease and the backbone carbonyl of the P1 residue resulting in the cleavage of the peptide bond. Protease specificity is determined by the P1-P1′ bond which is positioned such that it is readily accessible to proteases, Table 1 shows the residues involved in P1-P1′ in various serpins. RCL inserts into the *β* sheet A and transports the covalently bound protease with it. As a result protease gets translocated by over 70 Å (Figure 1) and its active site gets distorted [6, 8]. Distortion of the active site prevents the final hydrolysis events and the result is an irreversible covalent serpin-enzyme complex. When active serpins are proteolytically inactivated in a substrate-like reaction, they undergo an important structural transition with a resultant increase in their conformation stability [9]. The P4-P4′ sequence of RCL is highly conserved in all inhibitory serpins and mutations in this region (P2 Gly to Pro mutation in antithrombin) result in loss of inhibitory activity (Table 1). Furthermore, the amino acids of the hinge region have small side chain that allows loop flexibility necessary for complex formation. In contrast, the RCL of ovalbumin is in a fully extended, rigid alpha-helical conformation that is unable to conform to the active site of a protease which explains its lack of inhibitory activity. The driving force for this conformational change is thought to be the energy loss associated with the increased loop insertion in the complexed serpin.

## 3. Domains Involved in Serpin Inhibition Mechanism

**Figure 2:**
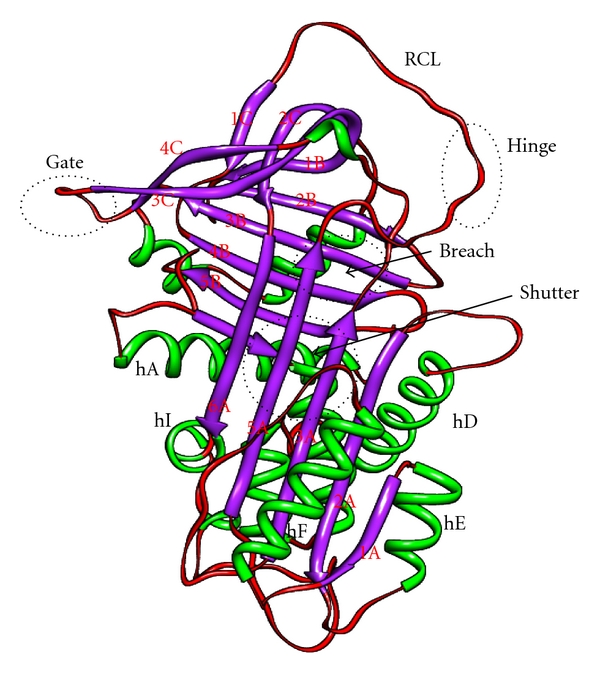
Important domains in serpin conformations. Several regions are important in controlling and modulating serpin conformational changes. The *Reactive Centre Loop* is involved in protease recognition and conformational transformation as strand 4A after inhibition. The P15–P9 portion of the RCL is called the *hinge* region. The point of initial insertion of the RCL which is the *breach* region, located at the top of the A *β*-sheet. Near the center of A *β*-sheet is the *shutter domain*. The breach and shutter are two major regions that assist sheet opening and accept the conserved hinge of the RCL when it inserts. The *gate region* is composed of s3C and s4C strands which has been primarily observed by studies of the transition latency. The image was drawn in chimera using the PDB file of native antitrypsin conformation.

Several regions are important in controlling and modulating serpin conformational changes; Figure 2 shows functionally and structurally important regions of serpin [10]. A portion of RCL from P15–P9 is called the hinge region [3, 11]. In S → R transition it provides mobility which is vital for the conformational change of the RCL. Hinge region contains many conserved residues between P15–P10, towards the N-terminal of the RCL. Out of these the amino acid at P14 (Ser-380) is of critical importance as its insertion in the *β*-sheet A is a prerequisite for inhibitory activity [12]. In antithrombin the replacement of P12 Ala by the Thr causes loss of inhibitory activity which is due to polymerization of antithrombin [13]. The breach region represents the point of initial insertion of the RCL which is located at the top of the A *β*-sheet [14]. Near the centre of A *β*-sheet is the shutter domain [15]. The breach and shutter are two major regions that assist sheet opening and accept the conserved hinge of the RCL when it inserts [8]. Highly conserved residues located in the shutter region of the *β*-sheet A of the serpin fold are Ser-53 and Ser-56 which play an important role in the serpin conformational transitions [16]. The gate region is composed of s3C and s4C strands which has been primarily observed by studies of the transition of active PAI-1 into latency [17]. In order to insert fully into the A *β*-sheet without cleavage, the RCL has to pass around the *β*-turn linking strand s3C and s4C [10, 18].

## 4. Ligand-Dependent Serpins

**Figure 3:**
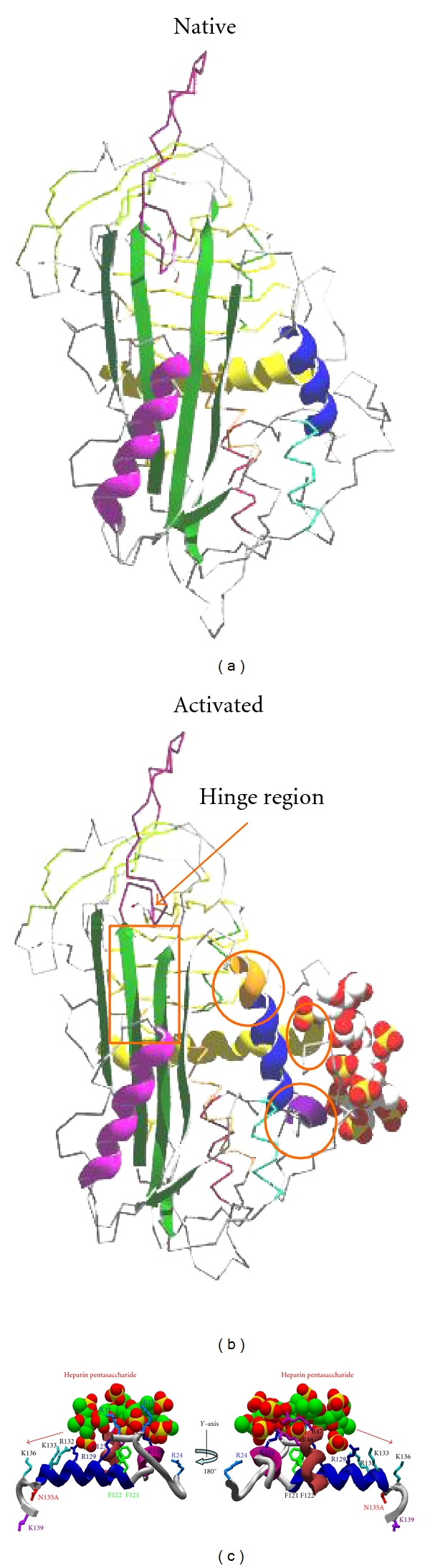
Conformational changes in cofactor (heparin) bound serpin (antithrombin) and residues involved in cofactor interaction. Heparin binding produces a series of conformational changes in antithrombin; extension of helix D by forming a 2 turn helix (P-helix) at the N-terminal end and a 1.5 turn extension of D-helix towards the C-terminal end. Moving of strand 3A and strand 5A and expulsion of reactive center loop leads to activated antithrombin. Given below are the basic residues in the heparin binding site that interact with the pentasaccharide are Lys-11 and Arg-13 in the N-terminal end; Arg-46 and Arg-47 in the A-helix; and Lys-114, Phe-121, Phe-122, Lys-125, and Arg-129 in the region of the D-helix. The figures were made by using antithrombin PDB (native 1E05; activated 1E03) files and swiss-prot PDB viewer.

One of the important features of serpins is their ability to bind various protease and nonprotease ligand (Table 2) and some of the examples of ligand-dependent serpins are antithrombin, heparin cofactor II, protein C inhibitor, plasminogen-activator inhibitor-1 (PAI-1), protease nexin-1 and kallistatin [19]. Binding of ligand to these serpins regulate their activity, like PAI-1 binds to vitronectin or ZPI binds to protein Z [20]. Several serpins which interact with glycosaminoglycan ligands have their reactions with proteases improved by such ligands in accordance with a ternary complex bridging mechanism. Vitronectin also seems to help the reaction of PAI-1 with thrombin [21]. A subfamily of serpins exists whose inhibitory activity is greatly accelerated upon binding of heparin and other negatively charged polyanions, such as heparan sulfate and dermatan sulphate. The members of this group include heparin cofactor II (HCII), antithrombin, protein C inhibitor, protease nexin 1 (PN-1) and PAI-1. Heparin is a highly negatively charged glycosaminoglycan consisting of alternating glucosamine and iduronic acid monomers. Studies have demonstrated that upon binding to heparin antithrombin gets activated. Binding of heparin pentasaccharide to the D helix of antithrombin causes a series of large conformational shifts (as shown in Figure 3). Extension of D-helix at both the ends leads to an interdomain rotation of the bottom half of the antithrombin relative to the top half, which leads to RCL expulsion from the *β*-sheet A and exposure of the P1 arginine (Arg-393) residue [18, 22]. The basic residues in this site that interact with the pentasaccharide are Lys-11 and Arg-13 in the N-terminal end; Arg-46 and Arg-47 in the A-helix; and Lys-114, Lys-125, and Arg-129 in the region of the D-helix (Figure 3). Two nonbasic residues, Phe-121 and Phe-122, reside near these positively charged amino acids of the heparin-binding domain but make minimal direct contact with the pentasaccharide sequence [23]. It has been shown that mutation of either Phe-121 or Phe-122 leads to decline in antithrombin-heparin binding affinity. Cofactor binding serpins are critically balanced to maintain its native metastability during its binding and transformation of conformational change (Figure 3).

## 5. Serpin Metastable Form

The metastable native state of serpins is thought to be like a kinetically trapped folding intermediate that is blocked by a very high kinetic barrier [24, 25]. The study of this kinetic trap provided an important link to understand structure-function relationships of serpins but the molecular basis by which this kinetic trap prevents the native serpin to form a more stable state is unknown [26]. It has been suggested that there are certain native interactions in the metastable form which drags it to get converted into a more stable form [24]. Studies have shown that the other stable conformation in the native serpin chain is the latent RCL-inserted form [17, 27]. For example the native form of a serpin, plasminogen activator inhibitor-1, readily gets transitioned into the latent form under physiological conditions with a half-life of 1-2 hours [28] and same happens in other serpins under mild denaturing conditions [29]. 

 In serpins, when RCL is inserted into the A *β*-sheet, there occur a gradual decline in the free energy of the serpin and an increase in its stability [30–32]. Surprisingly, serpins also undergo a rise in thermostability when complexed with peptides analogous to the RCL [33]. This suggests that increased stability is a product of hydrogen bond formation and hydrophobic interactions made by the addition of a sixth strand to the A *β*-sheet as s4A. But the negative consequence of obtaining metastability is that serpins are vulnerable to mutations that can result in aberrant structural rearrangement into dysfunctional states with increased stability [34].

## 6. The Molecular Basis of Serpin Metastability

Native state of the serpin is dependent on nonideal interactions (Table 3) which imparts strain within the native fold that can be further alleviated by adopting alternative conformations [35]. Nonideal interactions include the presence of hydrophobic pockets [36, 37], overpacking of side-chains [38], and the burial of polar groups [39] and cavities in the hydrophobic core of the protein [40]. Molecular details of how such structural defects control the protein functions are yet to be elucidated. Native conformation regulates the inhibitory function of *α*-1 antitrypsin by controlling the rate of the conformational switch during complex formation with a target protease. Hence the conformational switch is driven by mobilization of unfavourable interactions in the native state into more favourable ones, such interactions seem to have control over conformational switch [41]. From fluorescence studies it has been identified that nonnative interactions were residing around the top of the A *β*-sheet and the F-helix [42, 43]. Some unfavourable interactions that are involved in maintaining the metastable state of antitrypsin are summarized in Table 3. The unfavourable interactions made by residues of the F-helix might play a crucial role in preventing incorrect folding in serpin [35, 44]. Lys-335 is suitably situated near the F-helix to assist in the sheet A opening where it forms critical interaction with Ile-169 and Leu-172, a reduction in the side chain of these residues results in increase in stability which contributes in the native strain. Polar-nonpolar interactions also contribute to native stability, Phe-189 interacts with Gly-164 and Thr-165 in the F-helix providing unfavourable interaction which contributes in native metastability [40]. 

 Serpins make use of many conserved interactions which are distributed around the molecule to stay in the kinetic trap until a protease comes along and initiates further thermodynamically favourable conformational changes [44]. Decreasing the size of side chains in overpacked regions releases the native strain, and increasing the size of side chains in cavities stabilizes the native form, probably by providing better interactions with nearby residues. Likewise, filling exposed hydrophobic pockets by substituting larger residues also stabilizes the native state in antitrypsin [40]. Such unfavourable structural features are also found in other serpin proteins, like antichymotrypsin and antithrombin III, and the native strain in these serpins can also be relieved by compensating substitutions [45]. During protease inhibitor complex formation, unfavourable interactions in the native form are mobilized and transformed into more favourable ones [3]. These conformational changes result in the conversion of the metastable native form into a more stable conformation [46].

## 7. Serpin Polymerization

**Figure 4:**
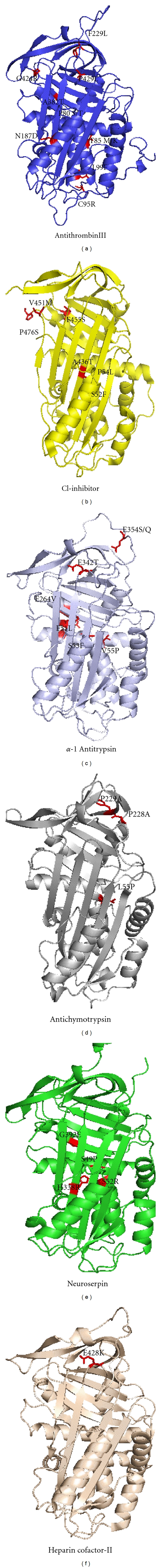
Point mutation in representative serpins that leads to latency, dimerization, aggregation and polymerization. Figure shows different serpin members like antithrombin (1e05), *α*-1 antitrypsin (2qug), neuroserpin (1jjo), antichymotrypsin (4caa), heparin cofactor-II (1jmj) and C1-inhibitor along with corresponding natural variant that gives rise to polymerization. Images were prepared by using Pymol visualization tool.

The tendency of the RCL of serpins to become an additional strand in a pre-existing *β*-sheet also makes them prone to other types of loop-sheet interactions [47]. As a protein family, serpins are particularly prone to the formation of stable polymers owing to the metastability of their native fold and the thermodynamically driven *β* sheet opening out mechanism required for their role as protease inhibitors [3, 6]. Approximately −32 kcal /mol energy is released by the incorporation of the reactive centre loop (RCL) of the serpin into the centre of its main *β*-sheet [48].  Figure 4 shows point mutation in some representative serpins that leads to dimerization, aggregation and polymerization. 

 Severe deficiency of the Z variant of *α*-1-antitrypsin (Glu342Lys) results from a conformational switch and forms a unique linkage between the reactive centre loop of one molecule and A *β* sheet of a second [49]. This process of polymer formation was dependent on temperature and concentration, and the polymers that were formed had the appearance of “beads on a string” when seen by electron microscopy [49, 50]. Since this initial report, investigations have been concerned with the characterization and classification of clinically relevant mutations. Neuroserpin is a member of the serine proteinase inhibitor (serpin) superfamily that is secreted from the growth cones of neurons and inhibits the enzyme tissue type plasminogen activator (tPA) [51]. Aberrant protein linkage in mutants of neuroserpin causes intracerebral inclusions and dementia. Four different mutations of neuroserpin (Ser49Pro, Ser52Arg, His338Arg and Gly392Glu) have been described in humans [52–54]. All four mutations cause the spontaneous formation of neuroserpin polymers that are retained as inclusion (or Collins) bodies within neurons in the deeper layers of the cortex and the substantia nigra [35, 55]. It can be responsible for the occurrence of dementia, tremor, seizures, epilepsy and dysarthria that is present in different degrees depending on the harshness of the mutation involved. In heparin cofactor-II, there are two known polymerization variants which are Glu428Lys and Pro433Leu [56].

## 8. Polymeric Conformations in Serpin

Loop A sheet polymeric structure is characterised by the insertion of RCL of one serpin molecule into A *β*-sheet of another between strand three and five [57]. Initial evidence of this intermolecular linkage came from studies in which *α*-1 antitrypsin polymerization was hindered by incubation with peptides analogous to the RCL [49, 58] that was further supported by fluorescence energy transfer data [59]. In loop C sheet polymer, one of the strand of C *β*-sheet moves away to allow full insertion of the RCL and the position left vacant by s1C is filled by the RCL of second molecule [35]. This polymeric form is found in Mmalton variant of antitrypsin, antithrombin can also forms similar type of polymer when heated in the presence of citrate [60]. s7A polymer has only been shown in PAI-1, which is characterised by hydrogen bonding of the RCL of one molecule with six strand of the *β*-sheet A of the other, where donor RCL become the seventh stand of the acceptor molecule's A *β*-sheet [61]. In disulfide linked polymeric form the RCL is not involved as a polymer formation domain as it does in other polymeric forms. Dimer *α*-1 antitrypsin has single cysteine residue located on the B *β*-sheet and this dimeric species goes on to form high order polymer. However, this disulfide-linked polymer has similar linear morphology to both loop A and C sheet as supported by electron microscopy [62].

## 9. Helix B Variants in the Shutter Region

The shutter region of serpins is comprised of a number of well-conserved residues that play a significant role in the maintenance of stability and function [35]. Mutations in shutter region allow the aberrant opening of the A-sheet, with the likelihood of the insertion into its lower half of the reactive loop of another molecule to give intermolecular linkage and polymerization of the serpin. Shutter region includes F-helix, B-helix, strand s3A and s5A of *β*-sheet A that plays an important role in stability and function in serpins [55]. Conserved helix B residues interact with the *β*-sheet A at the upper portion of the shutter region where RCL inserts as s4A. Helix B mutations in *α*-1-antichymotrypsin (Leu55Pro) and *α*-1-antitrypsin (Phe51Leu, Ser53Phe and Val55Pro) can cause lung (emphysema) and liver diseases (cirrhosis). Protein C-inhibitor (Ser52Phe and Ser54Leu) and antithrombin (Pro80Ser/Thr, Thr85Met/Lys, Cys95Arg and Leu99Phe) have mutations which can result in angioedema and thrombosis [5, 25, 35, 56]. Importance of strand 6B deformation and exposure of helix B in smooth insertion of the reactive centre loop during serpin inhibition was hypothesized recently and indicated that helix B exposure in variants may increase its polymerization propensity [63]. 

## 10. Accessible Surface Area and Stability Analysis of Serpin Variants

It is possible that the variants that contribute to serpin polymerization are deeply buried and introduce local destabilization. We took antithrombin, antitrypsin, antichymotrypsin, neuroserpin and heparin cofactor II as representative inhibitory serpins in which details of polymerization variants are known and did an analysis of burial and stability. Table 4 shows the positions of the serpin variants along with their ASA values. Analysis shows that in most cases the amino acid involved in the polymerization was completely buried in the native conformation. In the native state, antithrombin Pro-80, Thr-85, Leu-99, antitrypsin Phe-51, Ser-53 and Val-55 and neuroserpin Ser-49 and Ser-52 residues showed the ASA value of zero, these residues are part of helix B. Helix B residues tend to show the maximum burial especially the residues that are conserved in the serpin. 

 To test the effect of deeply buried helix B variant of serpin on the overall stability point mutations were done using I mutant 2.0 program at temp 25°C and pH 7.0 and the results are summarized in Table 4. The results showed that all the polymerization variants of the helix B were destabilizing with ΔΔG values ranging from −0.4 kcal/mole to −3.0 kcal/mole. No apparent correlation was found between the relative burial and the magnitude of destabilization. Most destabilized variants were not necessarily the most buried ones. However the helix B residues like Thr-85 in antithrombin, Val-55 in antitrypsin and Leu-55 in antichymotrypsin tend to be more destabilized in comparison to other polymerization variants in helix B. The result clearly indicates that the deeply buried residues can cause conformational flexibility which results in the global destabilization in the polymerizing variants of serpins. 

## 11. Conclusion

Serpins fold into structures that are metastable and employ a unique suicide substrate-like inhibitory mechanism to achieve stable state. Serpins make use of many conserved interactions like decreasing the size of side chains in overpacked regions, and increasing the size of cavities and filling of exposed hydrophobic pockets to release the native strain to convert from the native form to a cleaved inhibitory stable conformation. Due to its inhibition mechanism serpin are prone to conformational defects resulting in severe pathological disorders. Understanding the mechanism of polymer formation in serpins is confusing due to availability of several models and also due to the lack of crystal structures of natural variants. Identifying specific domains and interactions in serpin that contribute to inhibition and polymerization mechanism is important. Helix B region is a mutation hotspot in serpin that leads to polymerization due to deep burial, decreased stability and its involvement in RCL translocation.

## Figures and Tables

**Table 1:** Represents the Scissile bond in various serpins and their protease targets. Antithrombin is an efficient inhibitor of factor Xa, thrombin and factor IXa. Residues flanking the P1-P1′ are also critical for protease multispecificity.

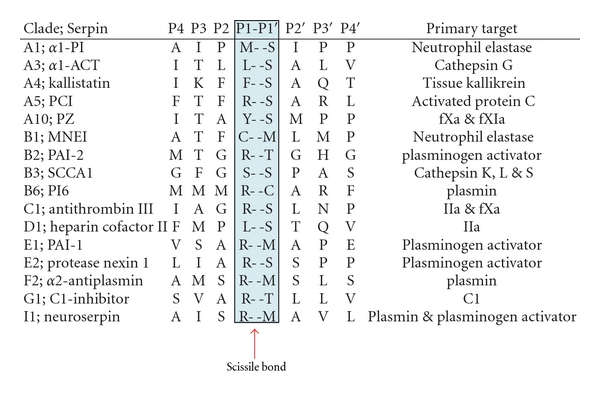

**Table 2:** Serpins and their known protease and nonprotease ligands.

Serpin	Non protease ligands	Protease target
Antithrombin	Heparin, heparin sulfate	Thrombin, factor Xa, factor IXa
Antichymotrypsin	DNA, A*β*_1–42 peptide_	Cathepsin G
Alpha-1 Proteinase inhibitor	—	Neutrophil elastase
C1-Inhibitor	Heparin, collagen	Cls of complement system
Headpin	—	Lysosomal cathepsin
Heparin CofactorII	Heparin, dermatan sulfate	Thrombin
HSP47	Collagen	—
Kallistatin	Heparin	Tissue Kallikerin
Maspin	Collagen	—
MENT	DNA	Nuclear cysteine proteinase
Plasminogen Activator inhibitor-1	Heparin, heparin sulfate, vitronectin	tPA, uPA, thrombin, aPC
Protein C inhibitor	Heparin, retinoic acid	uPA, thrombin, aPC
Protease nexin-1	Heparin, collagen	Thrombin uPA
Thyroxine Binding Globulin	Thyroxine, triiodothyronine	—
Protein Z dependent protease inhibitors (ZPI)	Protein Z	Factor Xa

**Table 3:** Unfavourable interactions that contribute to the metastability of the native antitrypsin. Nonideal interactions include the presence of hydrophobic pockets, overpacking of side-chains, the burial of polar groups, cavities in the hydrophobic core of the protein and polar nonpolar interactions [40]. Lys-335 is one of the residues in antitrypsin that has been shown to play a crucial role in conformational switch during the process of inhibition. Local strain due to Lys-335 interactions in the native state is critical for the inhibitory activity.

Over packing of side chains	Polar-nonpolar interactions	Cavity filling mutations	Favourable interactions
Lys-335	Phe-189—Gly-164	Gly164Val	Native (Lys-335 is buried)

Ile-169	Thr-165-Val-161	Ala183Val	Cleaved
	Thr-165-Ile-169		Lys-335 forms salt bridge with Asp-171

Leu-172	Leu-172-Asn-186	Thr114Phe	Ile-169
	Lys-331-Val-333	Gly117Phe	Lys-168-Glu-346 (salt bridge)

**Table 4:** Analysis of the residue burial and stability of natural variant of serpin involved in polymerization.

Serpins	ASA^b^	ΔΔG^c^
	(Native)	(kcal/mol)
Antithrombin-P80 S/T^a^	0.0	−0.97, −0.72
Antithrombin-T85 M/K^a^	2.9	−0.73, −3.04
Antithrombin-C95R	9.3	−1.33
Antithrombin-L99F	1.1	−1.03
Antithrombin-N187D	9.6	−1.92
Antithrombin-F229L	1.5	−0.92
Antithrombin-A382T	28.0	−0.33
Antithrombin-G424R	2.5	−1.30
Antithrombin-P429L	12.7	−0.18
Antitrypsin-F51L	0.0	−1.42
Antitrypsin-S53F	0.0	−0.42
Antitrypsin-V55P	2.6	−2.28
Antitrypsin-E264V	5.2	0.47
Antitrypsin-E342T	6.9	1.02
Neuroserpin-S49P	0.0	−1.19
Neuroserpin-S52R	0.0	−1.06
Neuroserpin-H338R	0.0	−1.37
Neuroserpin-G392E	0.0	−0.30
Antichymotrypsin-L55P	1.6	−2.17
Antichymotrypsin-P228A	4.9	−1.45
Heparin Cofactor-II-E428K	18.0	−0.22

^a^Two different variant at the same position.

^b^Accessible Surface Area (ASA) values were determined from DSSP algorithm. The pdb codes used for the analysis are as follows:** a**ntithrombin (1t1f), antitrypsin (1qlp), neuroserpin (1jjo and 3fgq), antichymotrypsin (1yxa), and heparin cofactor-II (1jmj).

^c^ΔΔG were determined for the variants by using Imutant 2.0 at pH 7.0 and 25°C, the values were determined by using the difference of ΔG between the wild-type and the polymerization variants mentioned in the table.
